# SIRT3-mediated deacetylation of PRDX3 alleviates mitochondrial oxidative damage and apoptosis induced by intestinal ischemia/reperfusion injury

**DOI:** 10.1016/j.redox.2019.101343

**Published:** 2019-10-12

**Authors:** Zhanyu Wang, Ruimin Sun, Guangzhi Wang, Zhao Chen, Yang Li, Yan Zhao, Deshun Liu, Huanyu Zhao, Feng Zhang, Jihong Yao, Xiaofeng Tian

**Affiliations:** a Department of Surgery, The Second Affiliated Hospital of Dalian Medical University, Dalian, 116023, China; b Department of Pharmacology, Dalian Medical University, Dalian, 116044, China

**Keywords:** PRDX3, SIRT3, Mitochondrial oxidative damage, Apoptosis, Intestinal ischemia reperfusion

## Abstract

**Background:**

Hydrogen peroxide (H_2_O_2_)-induced mitochondrial oxidative damage is critical to intestinal ischemia/reperfusion (I/R) injury, and PRDX3 is an efficient H_2_O_2_ scavenger that protects cells from mitochondrial oxidative damage and apoptosis. However, the function of PRDX3 in intestinal I/R injury is unclear. The aim of this study was to investigate the precise mechanism underlying the involvement of PRDX3 in intestinal I/R injury.

**Methods:**

An intestinal I/R model was established in mice with superior mesenteric artery occlusion, and Caco-2 cells were subjected to hypoxia/reoxygenation (H/R) for the *in vivo* simulation of I/R.

**Results:**

PRDX3 expression was decreased during intestinal I/R injury, and PRDX3 overexpression significantly attenuated H/R-induced mitochondrial oxidative damage and apoptosis in Caco-2 cells. The level of acetylated PRDX3 was clearly increased both *in vivo* and *in vitro*. The inhibition of SIRTs by nicotinamide (NAM) increased the level of acetylated PRDX3 and impaired the antioxidative activity of PRDX3. Furthermore, NAM did not increase the acetylation of PRDX3 in sirtuin-3 (SIRT3)-knockdown Caco-2 cells. Importantly, PRDX3 acetylation was increased in mice lacking SIRT3, and this effect was accompanied by serious mitochondrial oxidative damage, apoptosis and remote organ damage after intestinal I/R injury. We screened potential sites of PRDX3 acetylation in the previously reported acetylproteome through immunoprecipitation (IP) experiments and found that SIRT3 deacetylates K253 on PRDX3 in Caco-2 cells. Furthermore, PRDX3 with the lysine residue K253 mutated to arginine (K253R) increased its dimerization in Caco-2 cells after subjected to 12 h hypoxia and followed 4 h reoxygenation. Caco-2 cells transfected with the K253R plasmid exhibited notably less mitochondrial damage and apoptosis, and transfection of the K253Q plasmid abolished the protective effect of PRDX3 overexpression. Analysis of ischemic intestines from clinical patients further verified the correlation between SIRT3 and PRDX3.

**Conclusions:**

PRDX3 is a key protective factor for intestinal I/R injury, and SIRT3-mediated PRDX3 deacetylation can alleviate intestinal I/R-induced mitochondrial oxidative damage and apoptosis.

## 1 Introduction

Intestinal ischemia/reperfusion (I/R) is a life-threatening condition involving mucosal barrier damage and bacterial translocation that initially occurs in the intestine and is also a trigger for systemic inflammatory response syndrome (SIRS) and multiple organ dysfunction syndrome (MODS) [[1], [2], [3]]. The damage induced by intestinal I/R injury arises from not only interruption of the blood supply but also restoration of the blood supply, which is a more serious symptom that is accompanied by the mass production of reactive oxygen species (ROS) [[4], [5], [6], [7]]. Accumulated ROS, such as hydrogen peroxide (H_2_O_2_) and hydroxyl radical (·OH), modify intracellular molecules, damage cellular lipids, proteins and DNA, disrupt intestinal epithelial homeostasis, and ultimately induce apoptosis [8,9]. Most H_2_O_2_ is produced in mitochondria [10], where it also triggers mitochondrial oxidative damage. The disruption of mitochondrial function and integrity caused by mitochondrial oxidative damage impacts redox signaling and oxidative stress and thereby induces necrosis and apoptosis. Mitochondrial oxidative damage participates in a wide range of pathologies, such as liver fibrosis [11], atherosclerosis [12], diabetes mellitus [13], and I/R injury in the intestine [[5], [6], [7]], kidney [14,15] and heart [16]. Accordingly, the scavenging of mitochondrial H_2_O_2_ might be an important therapeutic measure for intestinal I/R injury.

Peroxiredoxins (PRDXs) are a family of effective thiol peroxidases comprising at least six isoforms in mammalian cells [17]. The isoforms PRDX1, PRDX2 and PRDX6 are localized in the cytoplasm, PRDX4 is found in the endoplasmic reticulum, PRDX5 is localized in peroxisomes and mitochondria, and PRDX3 is the major species in mitochondria [18]. Because PRDX3 is the most abundant and efficient H_2_O_2_-eliminating enzyme in mitochondria, it is an important mitochondrial antioxidant protein that serves as the target for nearly 90% of H_2_O_2_ produced in the matrix [19,20]. PRDX3 scavenges H_2_O_2_ through being oxidized into its inactive dimer form [21,22], and previous studies have reported that PRDX3 can effectively inhibit oxidative stress and apoptosis [23] and attenuate cellular damage [24]. Transgenic mice overexpressing PRDX3 exhibit reduced production of H_2_O_2_ in mitochondria and decreased oxidative damage compared with control mice [25]. We previously demonstrated that an increase in mitochondrial H_2_O_2_ is a pivotal pathological process of intestinal I/R injury [5]. Therefore, we hypothesize that PRDX3 protects the intestine from I/R injury by reducing mitochondrial H_2_O_2_ and apoptosis.

Sirtuin-3 (SIRT3), a highly conserved nicotinamide adenine dinucleotide (NAD^+^)-dependent deacetylase that is mainly expressed in mitochondria, regulates the function of several mitochondrial proteins involved in fatty acid oxidation, oxidative phosphorylation, and the antioxidant response system [[26], [27], [28]]. Furthermore, SIRT3 acts as the original deacetylase for more than 65% of all mitochondrial proteins [29], such as cyclophilin D (CypD) [30], manganese superoxide dismutase (MnSOD) [31], and isocitrate dehydrogenase (IDH2) [32]. Indeed, the global mitochondrial acetylproteome is markedly reprogrammed in SIRT3-knockout (KO) mice [29]. Because of its function in antioxidation, SIRT3 protects against severe tissue injury caused by cerebral [33], myocardial [34] and limb [35] I/R. However, SIRT3 cannot scavenge ROS immediately, and PRDX3 is modified by reversible acetylation [29,36]. Thus, we speculate that SIRT3 functions in intestinal I/R injury by deacetylating PRDX3.

The objectives of this study were to demonstrate the importance of PRD×3 in intestinal I/R injury, confirm the effect of PRDX3 acetylation on its activity, and explore the mechanism and function of SIRT3-mediated PRDX3 deacetylation in intestinal I/R injury.

## 2 Materials and methods

### 2.1 Experimental animals

Adult male C57BL/6 mice (aged 8 weeks) weighing 20 ± 2 g were obtained from the Laboratory Animal Center of Dalian Medical University (Dalian, China), and SIRT3-KO mice were purchased from the Cyagen Bioscience Incorporated Company (Guangzhou, China). All procedures were approved by the Institutional Ethics Committee of Dalian Medical University and conducted according to the Guidelines for the Care and Use of Laboratory Animals. Briefly, the model mice were subjected to superior mesenteric artery occlusion by a microvascular clamp for 45 min and then to 1, 2, 4 or 8 h of reperfusion [5,6]. Sham mice underwent the same protocol without vascular occlusion. The mice were sacrificed after the indicated period of reperfusion, and samples were harvested for analysis.

### 2.2 Human intestine samples

Human intestine samples were collected from patients at the Second Hospital of Dalian Medical University (Dalian, China). Informed consent forms were signed by all the patients, and this process was approved by the Ethics Committee of Dalian Medical University.

### 2.3 Histological analysis and TUNEL assay

Intestinal, lung, and liver tissues harvested from C57BL/6 mice were fixed and embedded in paraffin, sectioned at a thickness of 4 μm and stained with hematoxylin and eosin (H&E). Histopathological scores were assigned to the intestine, liver and lung based on the histological scoring systems described by Chiu [37], Mikawa [38] and Eckhoff [39], respectively. TUNEL staining was conducted with an *in situ* cell death measurement kit (Roche, Branchburg, NJ, USA) according to the manufacturer's instructions. Adherent cells were fixed with 4% paraformaldehyde, permeated with 0.3% Triton X-100 and sequentially stained with TUNEL and DAPI.

### 2.4 Lung MPO activity, caspase-3 activity assay and serum levels of TNF-α, IL-6, AST and ALT

The levels of serum tumor necrosis factor-α (TNF-α) and interleukin-6 (IL-6) were examined using specific ELISA kits (Proteintech, Wuhan, China) for mice according to the manufacturer's instructions. Serum alanine transaminase (ALT) and aspartate transaminase (AST) and lung myeloperoxidase (MPO) activity were examined using specific assay kits (Nanjing Jiancheng Corp., China) according to the manufacturer's instructions.

### 2.5 Caspase-3 activity assay

Caspase-3 activity was examined using a caspase-3 activity assay kit (Beyotime) according to the manufacturer's instructions. Briefly, caspase-3 catalyzes Ac-DEVD-pNA (acetyl-Asp-Glu-Val-Asp *p*-nitroanilide) into yellow *p*NA (*p*-nitroanilide), which is measured at 405 nm.

### 2.6 Cell culture, hypoxia/reoxygenation (H/R) and treatment

Caco-2 cells purchased from American Type Culture Collection (ATCC) were cultured in DMEM supplemented with 10% fetal bovine serum, 1% nonessential amino acids, 1% glutamine, and 1% penicillin and streptomycin and maintained in a humidified atmosphere with 5% CO_2_ at 37 °C. To mimic hypoxic conditions, the cells were incubated in a microaerophilic system (Thermo Fisher Scientific) with 5% CO_2_ and 1% O_2_ balanced with 94% N_2_ for 12 h. The cells were then cultured under normoxic conditions for reoxygenation. For nicotinamide (NAM) or trichostatin A (TSA) treatment, the cells were incubated with 10 mM NAM or 0.5 μM TSA [40,41] for 1–8 h or pretreated with 10 mM NAM for 6 h and then subjected to H/R.

### 2.7 siRNA transfection

Caco-2 cells were transfected with specific or control siRNA (GenePharma, Shanghai, China) using the Lipofectamine 3000 reagent (Invitrogen, Shanghai, China) for 48 h. The siRNA sense strand sequences were as follows: PRDX3, 5′-GCACUCUUGUCAGACUUAATT-3′ and 5′-UUAAGUCUGACAAGAGUGCTT-3’; SIRT3, 5′-GAAACUACAAGCCCAACGUTT-3′ and 5′-ACGUUGGGCUUGUAGUUUCTT-3’; and negative control, 5′-UUCUCCGAACGUGUCACGUTT-3′ and 5′-ACGUGACACGUUCGGAGAATT-3’ (GenePharma, Shanghai, China).

### 2.8 Plasmid construction and transient transfection

The PRDX3 and SIRT3 expression plasmids, as well as the PRDX3 mutant plasmid, were synthesized by GenePharma. Caco-2 cells were transfected with 2 μg of the PRDX3 or SIRT3 expression plasmid or the control vector using Lipofectamine 3000 for 48 h. The transfection procedures were conducted according to the manufacturer's recommended protocols.

### 2.9 Western blot analysis

Western blotting was conducted with primary antibodies against PRDX3 (Abcam, Ltd., Cambridge, UK), SIRT3 (Cell Signaling Technology, MA, USA), cleaved caspase-3 (Beyotime Institute of Biotechnology, Shanghai, China), TOMM20 (Cell Signaling Technology, MA, USA) and β-actin (Beyotime Institute of Biotechnology, Shanghai, China). Protein quantification was performed using Gel-Pro Analyzer version 4.0 (Media Cybernetics, MD, USA).

### 2.10 Coimmunoprecipitation (coIP) and IP

Total proteins were extracted in IP lysis buffer (20 mM Tris-HCl, 150 mM NaCl, and 1% Triton X-100, pH 7.5). For IP experiments, precleared lysates were incubated with equal amounts of anti-PRDX3 and Protein AG Magnetic Beads (Bimake, Selleck Chemical, Houston, TX, USA) according to the manufacturer's instructions. The precipitate was washed five times with washing buffer. The samples were then resuspended in 1 × loading buffer and boiled for 10 min. Finally, the supernatant was collected by magnetic separation, subjected to Western blotting with anti-SIRT3 or anti-acetyl lysine (Abcam Ltd., Cambridge, UK) as the primary antibody, and then incubated with anti-IgG heavy chain-specific secondary antibody (Abbkine Scientific Co., Ltd., USA).

### 2.11 Mitochondrial isolated and H_2_O_2_ examination

The mitochondria from intestinal samples and Caco-2 cells were isolated using a mitochondria isolation kit (TransGen Biotech, Beijing, China) based on the manufacturer's instructions. Briefly, Caco-2 cells were digested and centrifuged, and the pellets were washed with cold PBS, centrifuged, suspended with mitochondria isolation reagent, and incubated in an ice bath for 15 min. The suspension was homogenized and centrifuged at 600 g, and the supernatant was centrifuged in another tube at 11,000 g. The pellets were the isolated mitochondria, and the protein concentration of the isolated mitochondria was quantified using the BCA method. The mitochondrial H_2_O_2_ levels were examined using a hydrogen peroxide assay kit (Nanjing Jiancheng Corp., China) according to the manufacturer's recommended protocol. H_2_O_2_ reacts with molybdenic acid to form a stable complex, which is measured at 405 nm. Mitochondrial lysates from Caco-2 cells pretreated with excess catalase to remove H_2_O_2_ and H_2_O_2_ standard liquid were used as controls to calculate the content of H_2_O_2_.

### 2.12 Mito-SOX red and flow cytometry

The mitochondrial production of O_2_^−^ was determined using the mitochondrial superoxide indicator Mito-SOX Red (Invitrogen). Briefly, Caco-2 cells were plated on glass-bottomed vessels and incubated with 5 μM Mito-SOX Red for 10 min at 37 °C in the dark. The Mito-SOX Red fluorescence was visualized using a laser confocal microscope (Leica TCS SP5II, Germany). Caco-2 cells stained with Mito-SOX Red were also analyzed by flow cytometry using a BD Biosciences FACSCalibur flow cytometer.

### 2.13 Immunofluorescence staining

Caco-2 cells treated with 200 nM MitoTracker CMXRos (Invitrogen) were cultured in a glass-bottomed vessel for 30 min at 37 °C protected from light, washed with PBS, fixed in 4% PFA for 30 min at room temperature, and treated with 0.1% Triton X-100 in PBS for 10 min for permeation. The cells were then washed with PBS, blocked with 1% BSA for 1 h at 37 °C, and incubated with anti-PRDX3 antibody overnight at 4 °C. The cells were then washed again, incubated with Alexa Fluor 488-conjugated secondary antibody (Proteintech) for 1 h at room temperature, and washed with PBS. The nuclei were stained using a DAPI solution for 5 min at room temperature, and the cells were subsequently visualized under a laser confocal microscope (Leica TCS SP5II, Germany).

### 2.14 Mitochondrial membrane potential

The mitochondrial membrane potential was examined using a JC-1 probe (Beyotime Institute of Biotechnology, Shanghai, China) according to the manufacturer's instructions [42]. Briefly, JC-1 gathered into J-aggregates in the mitochondrial matrix and showed red fluorescence under conditions of a higher mitochondrial membrane potential, whereas the JC-1 monomer showed a green fluorescence when the mitochondrial membrane potential was lower. The mitochondrial membrane potential of isolated mitochondria was examined using a fluorescence spectrophotometer with an excitation wavelength of 490 nm and an emission wavelength of 530 nm for the JC-1 monomer with an excitation wavelength of 525 nm and an emission wavelength of 590 nm for the JC-1 aggregates. The ratio of red to green JC-1 fluorescence was calculated.

### 2.15 Transmission electron microscopy (TEM)

The mitochondrial morphology was examined by TEM. Caco-2 cells were fixed and embedded, and ultrathin sections were cut at a thickness of 50 μM, dehydrated in a gradient ethanol series and embedded in epoxy resin. The samples were then stained with uranyl acetate and lead citrate and observed using a transmission electron microscope (JEOL, Peabody, MA).

### 2.16 Statistical analysis

All the data are expressed as the means ± standard deviations (SDs), and the data analyses were performed using GraphPad Prism software (version 5.0; GraphPad Prism Software, La Jolla, CA, USA). The data were analyzed to determine the statistical significance of the differences between groups. *p* < 0.05 was considered significant.

## 3 Results

### 3.1 PRDX3 alleviates mitochondrial oxidative damage and apoptosis in intestinal I/R

**Fig. 1:**
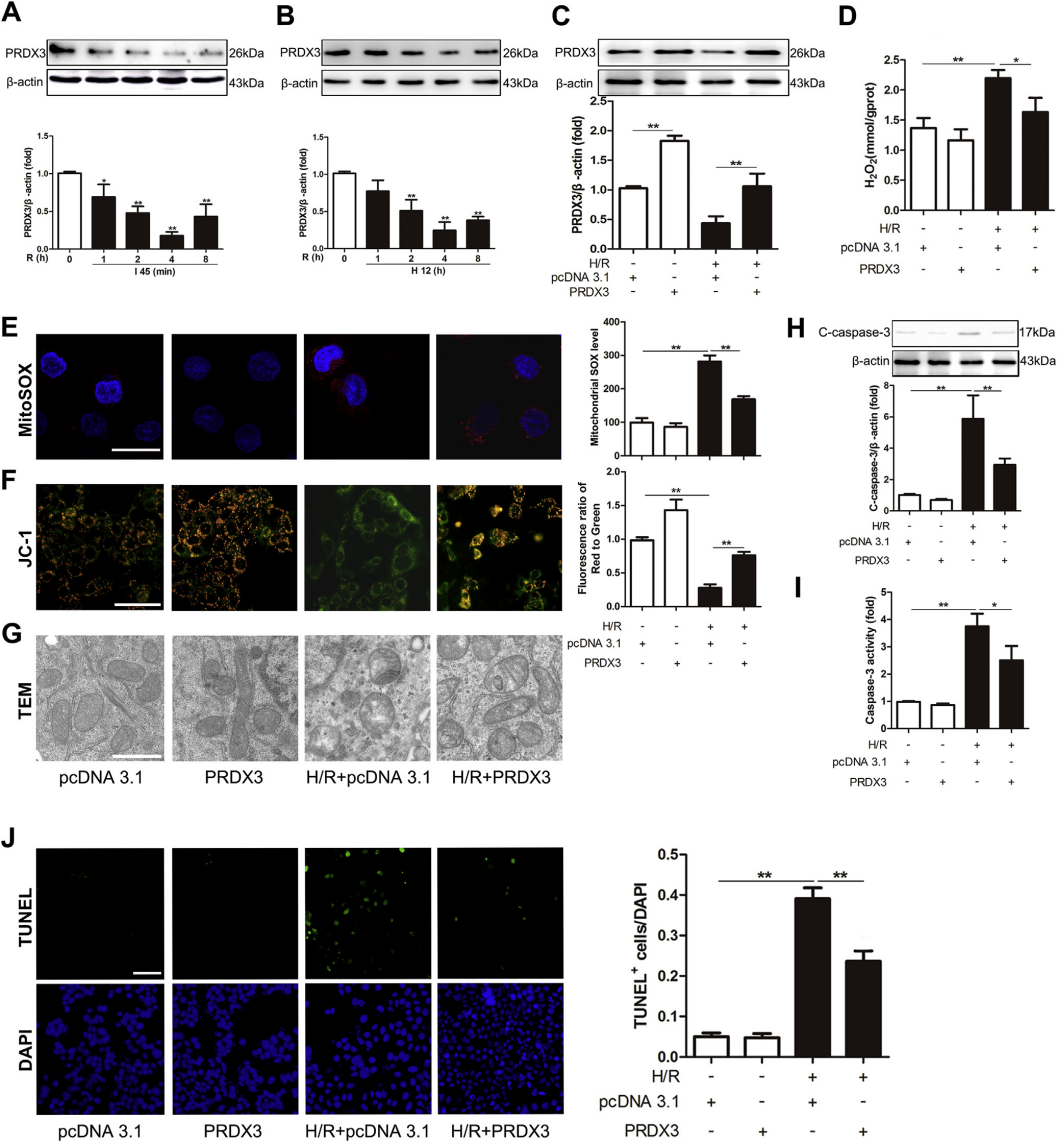
**PRDX3 alleviates mitochondrial oxidative damage and apoptosis in intestinal I/R**. (A) Mice were subjected to intestinal ischemia for 45 min followed by 1–8 h of reperfusion. Representative Western blot of PRDX3 monomer after 1–8 h of reperfusion. I, ischemia; R, reperfusion; n = 6. *p < 0.05 compared with the Sham group; **p < 0.01 compared with the Sham group. (B) Caco-2 cells were subjected to 12 h of hypoxia followed by 1–8 h of reoxygenation. PRDX3 monomer expression was decreased after 1–8 h of reperfusion. H, hypoxia; R, reoxygenation; n = 6. **p < 0.01 compared with the control group. (C–J) Caco-2 cells were transfected with the PRDX3 expression plasmid or vector plasmid for 48 h and then subjected to 12 h of hypoxia followed by 4 h of reoxygenation to achieve H/R. (C) PRDX3 monomer expression was increased after transfection with the PRDX3 expression plasmid, n = 3. (D–J) The protective effect of PRDX3 on mitochondrial damage and apoptosis was measured. (D) Mitochondrial H_2_O_2_ levels, n = 8. (E) MitoSOX Red staining and flow cytometry analysis of cells stained with MitoSOX dyes. Scale bar = 25 μm, n = 6. (F) The mitochondrial membrane potential was measured by JC-1, and the ratio of red to green fluorescence in isolated mitochondria stained with JC-1 probes was calculated. Scale bar = 50 μm, n = 6. (G) The mitochondrial morphology was determined by TEM. Scale bar = 10 μm. (H) Representative immunoblot of cleaved caspase-3, n = 3. (I) Caspase-3 activity in Caco-2 cells, n = 8. (J) TUNEL and DAPI staining. The apoptotic index is represented as the ratio of TUNEL-positive cells to DAPI staining. Scale bar = 50 μm, n = 6. *p < 0.05, **p < 0.01. (For interpretation of the references to colour in this figure legend, the reader is referred to the Web version of this article.)

To determine the role of PRDX3 after intestinal I/R injury, the expression of PRDX3 monomer at different times after reperfusion was evaluated. The results showed that PRDX3 monomer was clearly decreased after 45 min of ischemia followed by 1–4 h of reperfusion, particularly at 4 h, and then gradually increased during the remaining 8 h of reperfusion (Fig. 1A). Caco-2 cells were subjected to H/R injury *in vitro*, and the results were the same as those observed *in vivo* (Fig. 1B). To further determine the role of PRDX3 *in vitro*, a PRDX3 expression plasmid was transfected into Caco-2 cells (Fig. 1C). As expected, the overexpression of PRDX3 clearly reduced the H/R-induced mitochondrial generation of H_2_O_2_ in Caco-2 cells compared with that observed in the H/R group (Fig. 1D). Consistently, PRDX3 dramatically reduced H/R-induced mitochondrial O_2_^−^ production, as indicated by the MitoSOX Red experiment and flow cytometry analysis (Fig. 1E). Furthermore, we measured the mitochondrial membrane potential in Caco-2 cells and isolated cell mitochondria [43]. H/R injury induced obvious mitochondrial dysfunction, as indicated by the increased amount of JC-1 monomer showing green fluorescence. PRDX3 overexpression significantly improved mitochondrial function, as shown by the formation of JC-1 aggregates with red fluorescence (Fig. 1F). In addition, PRDX3 markedly alleviated mitochondrial swelling and the breaking of cristae induced by H/R injury in Caco-2 cells (Fig. 1G). Accordingly, PRDX3 overexpression reduced apoptosis, as indicated by decreases in the cleaved caspase-3 level and activity and a reduced number of TUNEL-positive cells compared with those found in the H/R group (Fig. 1H-1J). Thus, our study demonstrates that a reduction in PRDX3 expression is a critical pathological mechanism during intestinal I/R and that the overexpression of PRDX3 can alleviate H/R-induced mitochondrial oxidative damage and apoptosis.

### 3.2 PRDX3 acetylation plays a vital role in intestinal I/R injury

**Fig. 2:**
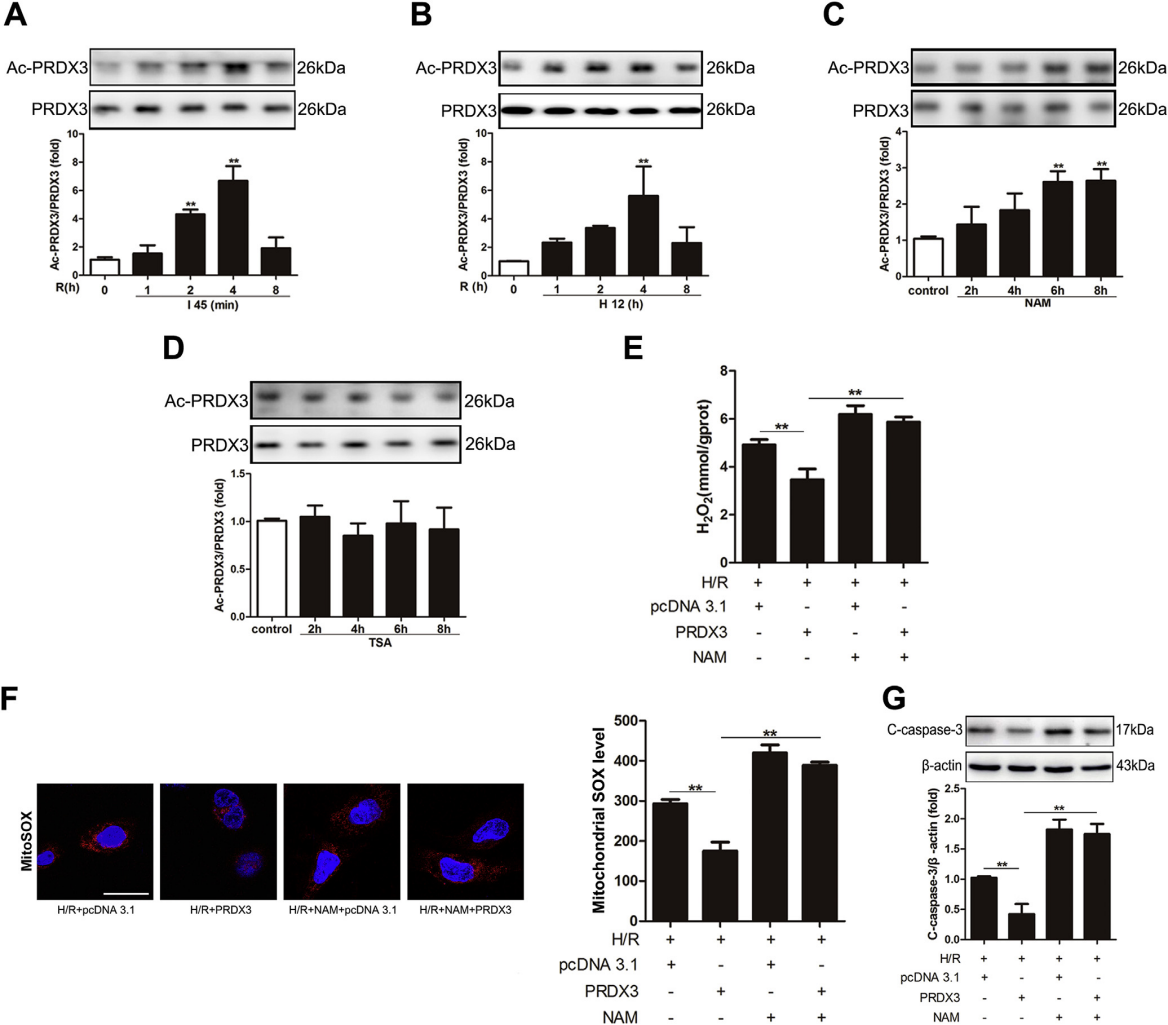
**PRDX3 acetylation plays a vital role in intestinal I/R injury**. (A) PRDX3 acetylation in the intestine subjected to 45 min of intestinal ischemia followed by 1–8 h of reperfusion, n = 6. (B) PRDX3 acetylation in Caco-2 cells after reoxygenation for 1–8 h, n = 6. (C) PRDX3 acetylation in Caco-2 cells after treatment with NAM for 2–8 h, n = 6. (D) PRDX3 acetylation in Caco-2 cells after treatment with TSA for 2–8 h, n = 6. (E–G) Caco-2 cells were pretreated with NAM and/or transfected with the PRDX3 expression plasmid and then subjected to H/R. (E) Mitochondrial H_2_O_2_ level, n = 8. (F) MitoSOX Red staining and flow cytometry analysis of cells stained with MitoSOX dyes. Scale bar = 25 μm, n = 6. (G) Representative immunoblot of cleaved caspase-3 in Caco-2 cells, n = 3. *p < 0.05, **p < 0.01. (For interpretation of the references to colour in this figure legend, the reader is referred to the Web version of this article.)

Reversible acetylation might influence PRDX3 activity against ROS [29]. To determine whether PRDX3 acetylation is induced in intestinal I/R injury, we examined the acetylation level of PRDX3 after reperfusion by IP *in vivo* and *in vitro*. The PRDX3 acetylation levels were increased after 2 and 4 h of reperfusion, particularly after 4 h (Fig. 2A), and these results are identical to those observed *in vitro* (Fig. 2B). Furthermore, the SIRT inhibitor NAM increased the acetylation level of PRDX3 (Fig. 2C), but the histone deacetylase (HDAC) inhibitor TSA did not induce a similar increase (Fig. 2D). This finding suggests that PRDX3 deacetylation is catalyzed by NAD^+^-dependent deacetylase. To further clarify the role of acetylation in regulating PRDX3 activity, we ectopically expressed PRDX3 in Caco-2 cells, and the cells were then treated with NAM before H/R. NAM exacerbated H/R-induced mitochondrial ROS and apoptosis and inhibited the effect of PRDX3 on mitochondrial ROS (Fig. 2E-2F) and apoptosis (Fig. 2G). Thus, we demonstrated that PRDX3 acetylation is vital to intestinal I/R injury.

### 3.3 PRDX3 is deacetylated by SIRT3 in the intestine

**Fig. 3:**
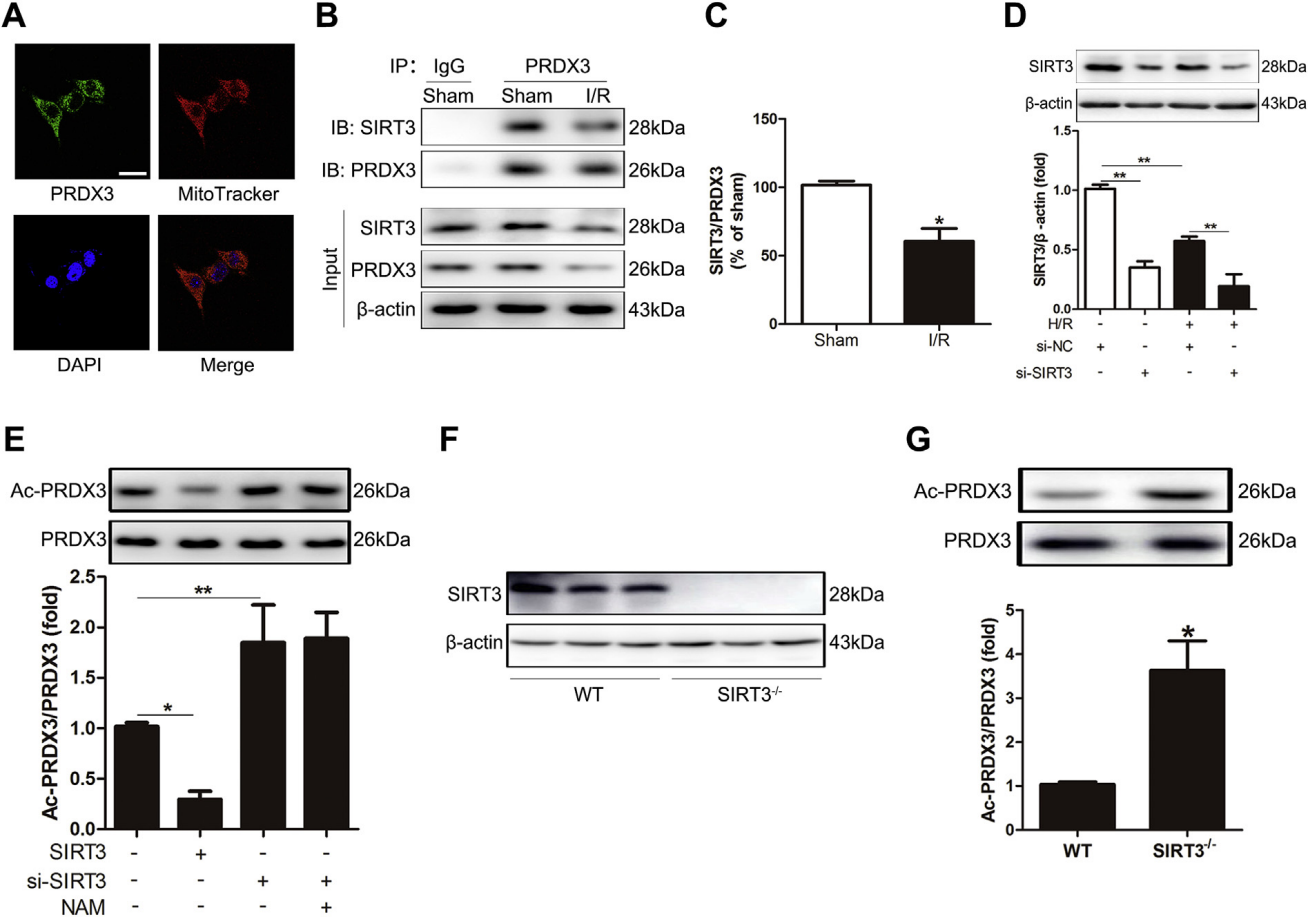
**PRDX3 is deacetylated by SIRT3 in the intestine**. (A) Localization of PRDX3 and mitochondria (MitoTracker). Scale bar = 25 μm, n = 6. (B, C) CoIP of PRDX3 with SIRT3 from the intestines of sham mice and mice after 4 h of reperfusion. The input represents the total protein extract used in IP. IB, immunoblotting; IP, immunoprecipitation; IgG, negative control, n = 3. (D) SIRT3 protein expression was normalized to β-actin in Caco-2 cells, n = 3. (E) PRDX3 acetylation in Caco-2 cells pretreated with NAM or/and si-SIRT3, n = 3. (F) SIRT3 protein expression in the intestines of SIRT3 WT mice and SIRT3-KO mice, n = 3. (G) PRDX3 acetylation in the intestines of SIRT3 WT mice and SIRT3-KO mice, n = 3. *p < 0.05, **p < 0.01.

Because the acetylation of PRDX3 was shown to be significant, we further explored the NAD^+^-dependent deacetylase that deacetylates PRDX3 in mitochondria. PRDX3 was mainly located in mitochondria (Fig. 3A), as confirmed by immunofluorescence analysis and the MitoTracker probe experiment. Because SIRT3 is the major mitochondrial deacetylase, we examined the relationship between SIRT3 and PRDX3 in mitochondria. CoIP experiments showed that SIRT3 interacted with PRDX3 (Fig. 3B). Remarkably, their interaction was substantially decreased under intestinal I/R conditions (Fig. 3C). We subsequently examined whether this interaction impacts the acetylation of PRDX3 in cells after SIRT3 knockdown (Fig. 3D) and overexpression. The overexpression of SIRT3 reduced the acetylation level of PRDX3. In contrast, SIRT3 knockdown significantly increased the acetylation of PRDX3. However, NAM treatment did not change the PRDX3 acetylation levels in SIRT3-knockdown cells (Fig. 3E). Importantly, the acetylation level of PRDX3 in SIRT3-KO mice was clearly increased compared with that in the WT group (Fig. 3F-3G). These results suggest that SIRT3 is the major NAD^+^-dependent deacetylase that deacetylates PRDX3 in the intestine.

### 3.4 SIRT3 protects against intestinal I/R injury both *in vivo* and *in vitro*

**Fig. 4:**
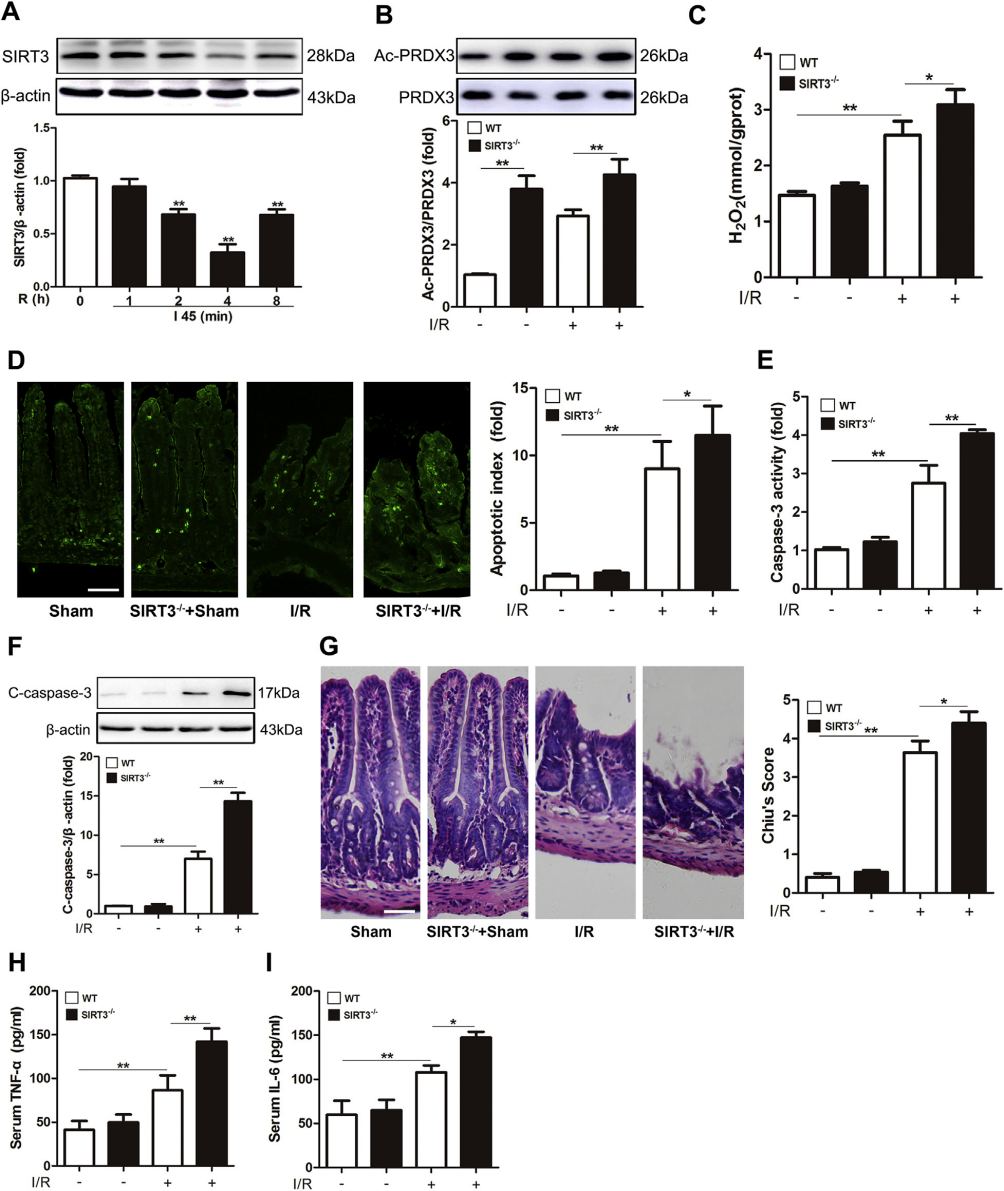
**SIRT3 protects against intestinal I/R injury *in vivo***. (A) Representative Western blot of PRDX3 after 1–8 h of reperfusion, n = 3. **p < 0.01 compared with the Sham group. (B–I) Mice were subjected to 45 min of intestinal ischemia followed by 4 h of reperfusion. (B) PRDX3 acetylation in SIRT3-KO mice and WT mice, n = 3. (C) Mitochondrial H_2_O_2_ level in the intestine, n = 8. (D) TUNEL staining and apoptosis index. Scale bar = 100 μm, n = 8. (E) Caspase-3 activity in the intestine, n = 8. (F) Cleaved caspase-3 protein expression was normalized to β-actin in tissue lysates from the mouse intestine, n = 3. (G) H&E staining and Chiu's score. Scale bar = 100 μm, n = 8. (H) Serum TNF-α, n = 8. (I) Serum IL-6, n = 8. *p < 0.05, **p < 0.01.

To study the function of SIRT3 in intestinal I/R injury, we examined the expression of SIRT3 in the mouse intestine. SIRT3 expression was evidently decreased after 2 and 4 h of reperfusion and markedly increased after 8 h of reperfusion, and the lowest SIRT3 expression was observed after 4 h of reperfusion (Fig. 4A). The function of SIRT3 in intestinal I/R injury was verified using SIRT3-KO mice. SIRT3 KO remarkably increased the acetylation level of PRDX3 compared with that in WT mice (Fig. 4B).

**Supplementary Fig. 1:**
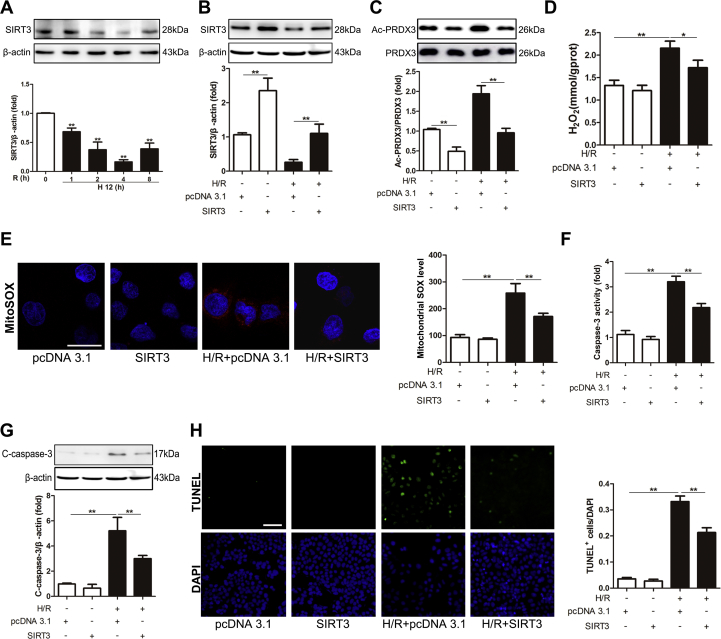


Furthermore, the intestines from SIRT3-KO mice showed more serious mitochondrial oxidation damage (Fig. 4C), apoptosis (Fig. 4D-4F) and histological injury (Fig. 4G) after I/R injury compared with the intestines from WT mice. The IL-6 and TNF-α levels were also increased in SIRT3-KO mice after intestinal I/R injury (Fig. 4H-4I). However, we observed the opposite trend following SIRT3 overexpression *in vitro* (Supplementary Figs. 1A–1H). These findings suggest that SIRT3 protects the intestine from I/R injury.

### 3.5 SIRT3 KO aggravates intestinal I/R-induced remote organ injury

**Fig. 5:**
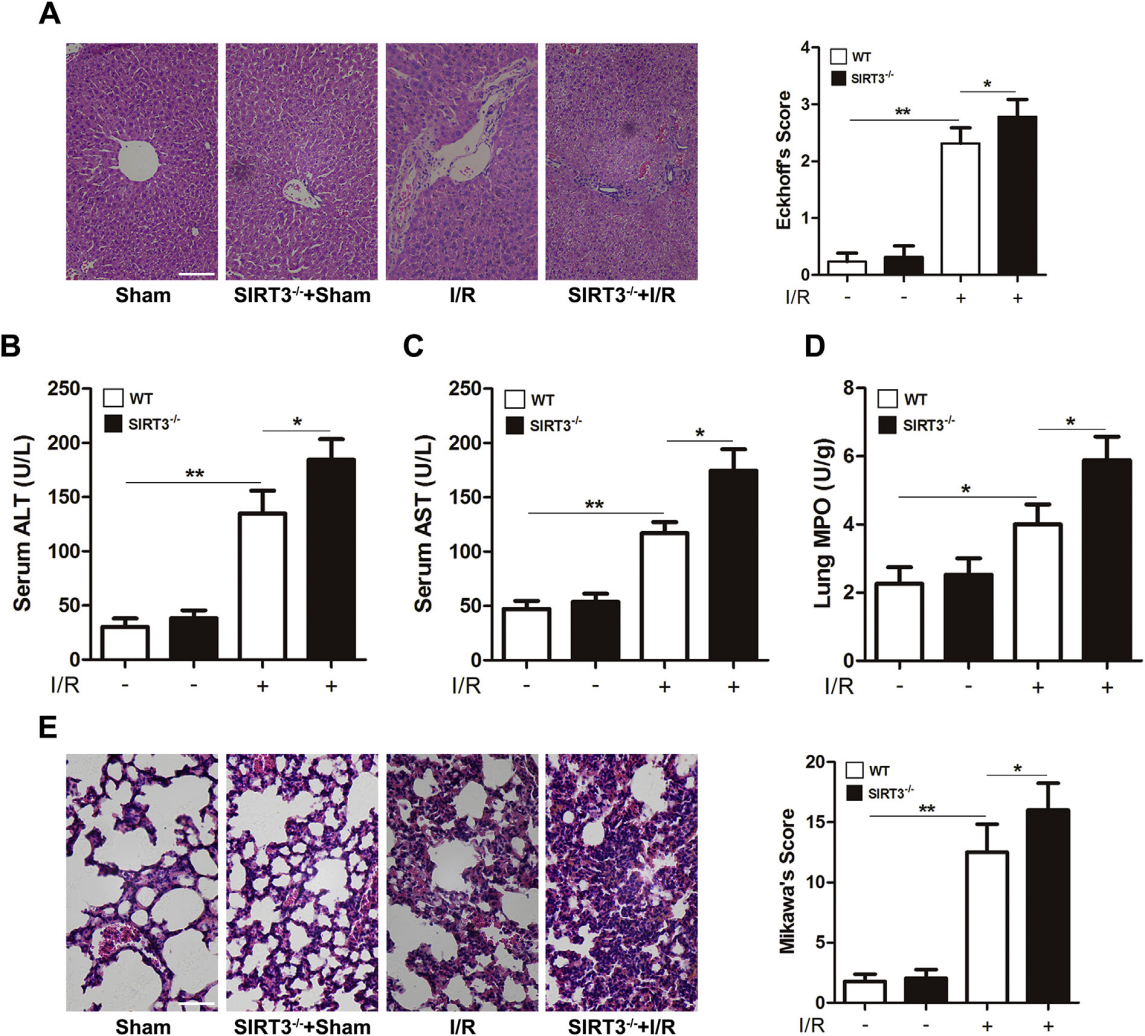
**SIRT3 KO aggravates intestinal I/R-induced remote organ injury**. (A) Liver H&E staining and Eckhoff's score. Scale bar = 200 μm, n = 8. (B) Serum ALT, n = 8. (C) Serum AST, n = 8. (D) Lung MPO activity, n = 8. (E) Lung H&E staining and Mikawa's score. Scale bar = 100 μm, n = 8. *p < 0.05, **p < 0.01.

Intestinal I/R not only injures the intestine but also seriously damages remote organs [[44], [45], [46]]. We thus examined the injury to the liver and lung after intestinal I/R. As shown in Fig. 5A, SIRT3 KO obviously exacerbated intestinal I/R-induced liver histological injury and increased the ALT and AST levels compared with those in SIRT3 WT mice (Fig. 5B-5C). Similarly, SIRT3 KO aggravated intestinal I/R-induced lung neutrophilic infiltration (Fig. 5D) and histological injury (Fig. 5E). These results indicate that SIRT3 KO aggravates intestinal I/R-induced liver and lung injury.

### 3.6 The function of SIRT3 in intestinal I/R injury partially depends on PRDX3

**Fig. 6:**
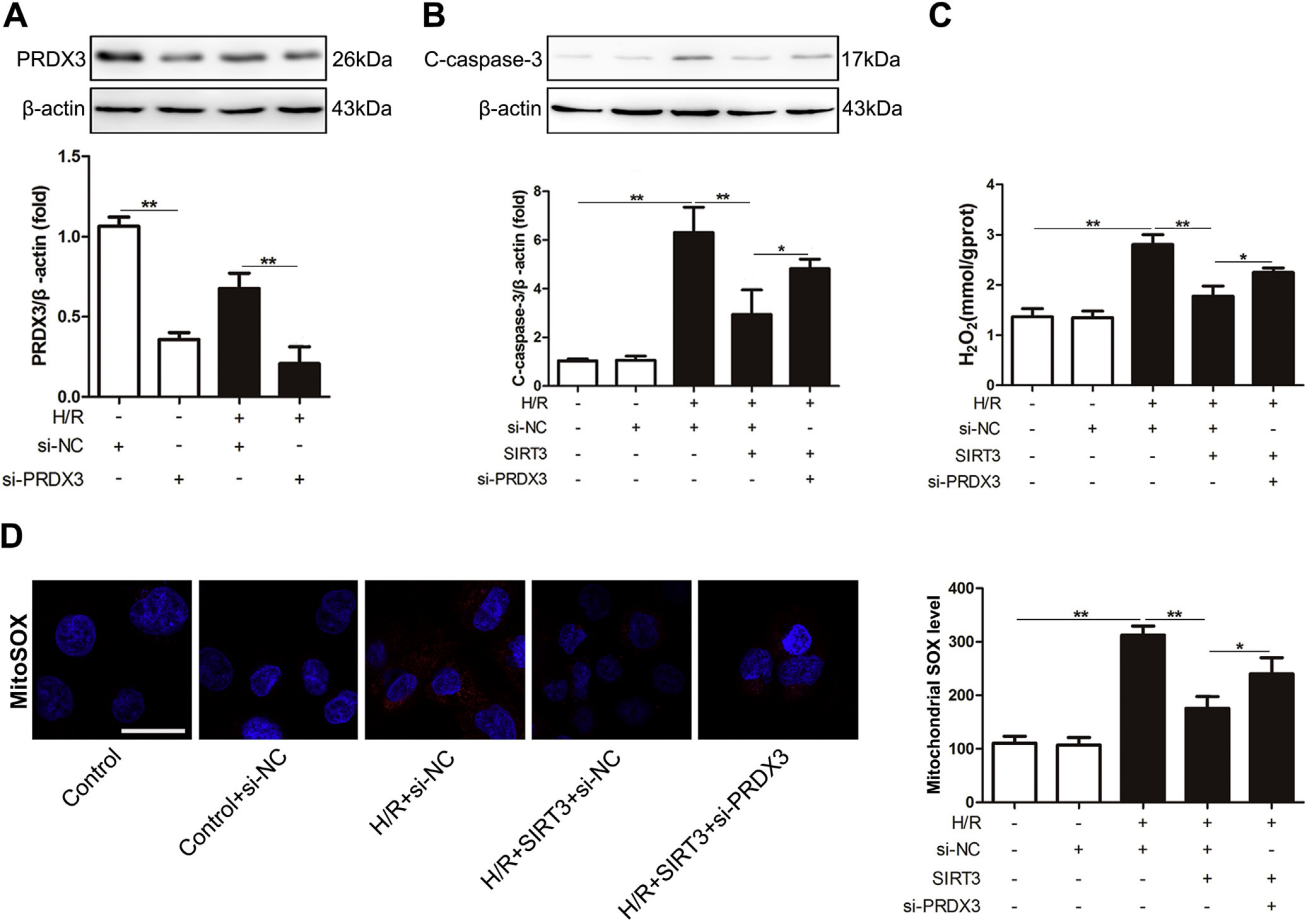
**The function of SIRT3 in intestinal I/R injury partially depends on PRDX3**. (A) The PRDX3 and (B) cleaved caspase-3 protein expression levels were normalized to β-actin expression in total cell lysates from Caco-2 cells, n = 3. (C) Mitochondrial H_2_O_2_ level, n = 8. (D) MitoSOX Red staining and flow cytometry analysis of cells stained with MitoSOX dye. Scale bar = 25 μm, n = 6. *p < 0.05, **p < 0.01. . (For interpretation of the references to colour in this figure legend, the reader is referred to the Web version of this article.)

To confirm whether PRDX3 is a downstream mediator of SIRT3, Caco-2 cells were cotransfected with SIRT3 plasmid and PRDX3 siRNA prior to being subjected to H/R. As expected, the protective effect of SIRT3 against mitochondrial ROS and apoptosis in intestinal I/R was reduced following PRDX3 knockdown (Fig. 6A-D), which indicated that the protective effect of SIRT3 in intestinal I/R injury is partially dependent on PRDX3.

### 3.7 SIRT3 deacetylates PRDX3 at K253 in Caco-2 cells

**Fig. 7:**
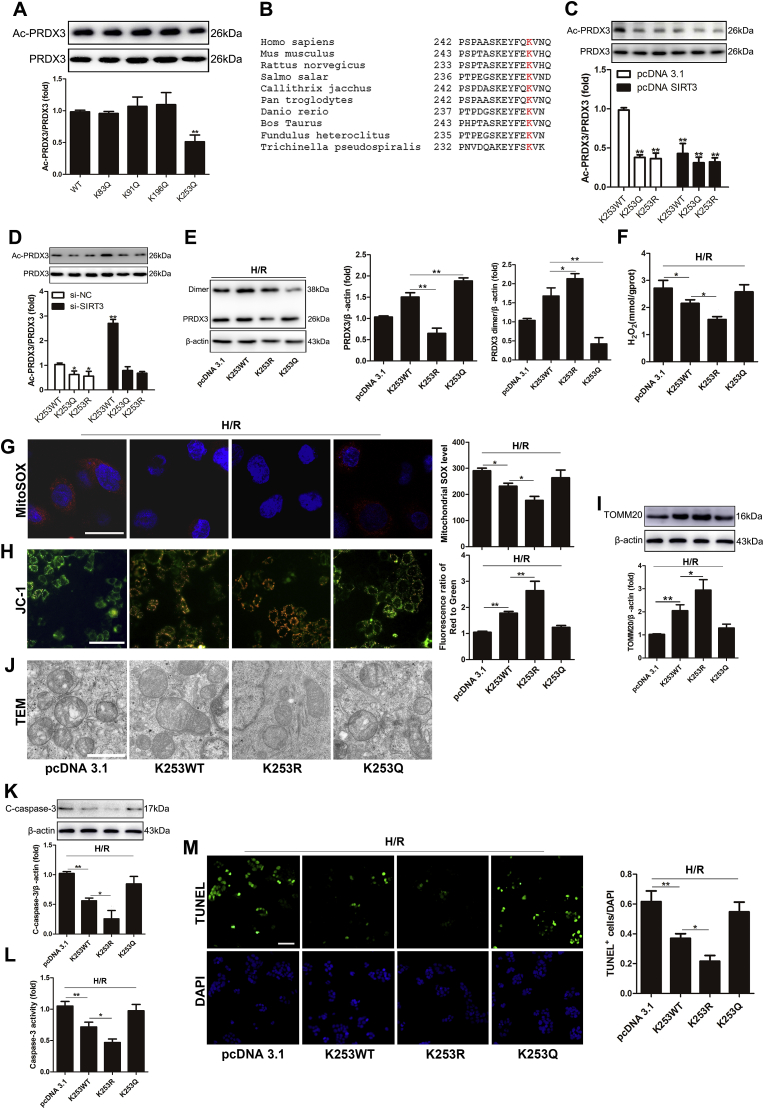
**SIRT3 deacetylates PRDX3 at K253 in Caco-2 cells**. (A) PRDX3 acetylation in Caco-2 cells transfected with potential acetylation site-specific mutants of PRDX3, n = 3. (B) Partial amino acid fragment of PRDX3 in several species. (C, D) PRDX3 acetylation, n = 3. (E–L) Caco-2 cells were transfected with no-load plasmid, PRDX3 expression plasmid, PRDX3 K253R or K253Q plasmid and then subjected to H/R injury; these cells groups are denoted pcDNA 3.1, K253WT, K253R and K253Q, respectively. (E) Representative Western blot of PRDX3 monomer and dimer in Caco-2 cells H/R injury. (F) Mitochondrial H_2_O_2_ level, n = 8. (G) MitoSOX Red and flow cytometry analysis of cells stained with MitoSOX dye. Scale bar = 25 μm, n = 6. (H) The mitochondrial membrane potential was measured by JC-1, and the ratio of red to green fluorescence ratio in isolated mitochondria stained with JC-1 probes was calculated. Scale bar = 50 μm, n = 6. (I) The mitochondrial mass was measured by immunoblotting of TOMM20, n = 3. (J) The mitochondrial morphology was determined by TEM. Scale bar = 10 μm. (K) The expression of cleaved caspase-3 protein was normalized to β-actin expression in total cell lysates from Caco-2 cells, n = 3. (L) Caspase-3 activity in Caco-2 cells, n = 8. (M) TUNEL and DAPI staining. The apoptotic index is represented as the ratio of TUNEL-positive cells to DAPI staining. Scale bar = 50 μm, n = 6. *p < 0.05, **p < 0.01. . (For interpretation of the references to colour in this figure legend, the reader is referred to the Web version of this article.)

**Supplementary Fig. 2:**
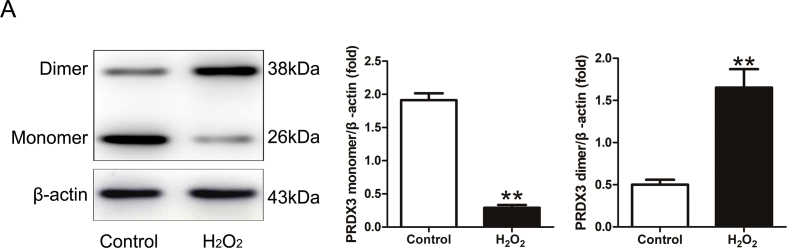


We further determined the SIRT3-targeted deacetylation sites on intestinal PRDX3. K83, K91, K196 and K253 are potential acetylation sites (Supplementary Table 1) on PRDX3 in humans [29,36]. We transfected Caco-2 cells with individual site-specific mutant plasmids and then examined the acetylation levels of PRDX3. Transfection with only the K253Q plasmid markedly reduced the acetylation level of PRDX3 (Fig. 7A), and this lysine residue is highly conserved among species (Fig. 7B). In contrast, neither SIRT3 overexpression (Fig. 7C) nor SIRT3 knockdown (Fig. 7D) altered the acetylation level of PRDX3 in Caco-2 cells transfected with K253Q or K253R, which suggested that K253 might be the main target of SIRT3 on PRDX3 in Caco-2 cells. PRDX3 scavenges H2O2 through being oxidized into its dimer form [21,22]. Here, we confirmed that Caco-2 cells treated with H_2_O_2_ caused the dimerization [47,48] of PRDX3 (Supplementary Fig. 2A). Furthermore, the mutation of the K253 lysine residue in PRDX3 to arginine (K253R) in Caco-2 cells clearly increased the dimerization of PRDX3 induced by H/R injury. In contrast, an acetylation mimic (mutation of lysine to glutamine, K253Q) decreased the dimerization of PRDX3 (Fig. 7E) in Caco-2 cell H/R injury. The overexpression of PRDX3-K253R resulted in notably lower mitochondrial H_2_O_2_ (Fig. 7F) and superoxide (Fig. 7G) production. In addition, PRDX3-K253R rescued mitochondrial dysfunction (Fig. 7H) and increased the mitochondrial mass (Fig. 7I), as confirmed by immunoblotting of the mitochondrial-specific protein TOMM20 [49]. In addition, in Caco-2 cells, PRDX3-K253R more obviously restored the morphological damage to the mitochondria (Fig. 7J) and alleviated apoptosis (Fig. 7K–7M) compared with the results observed with K253WT. A functional mimic of deacetylation increased the protective effect of PRDX3 against Caco-2 cell H/R injury. And K253Q abolished the protective effect of PRDX3 overexpression. These results suggest that SIRT3 deacetylates and activates PRDX3 mainly through the K253 residue.

### 3.8 Expression of SIRT3 and PRDX3 in the ischemic intestine of clinical patients

**Fig. 8:**
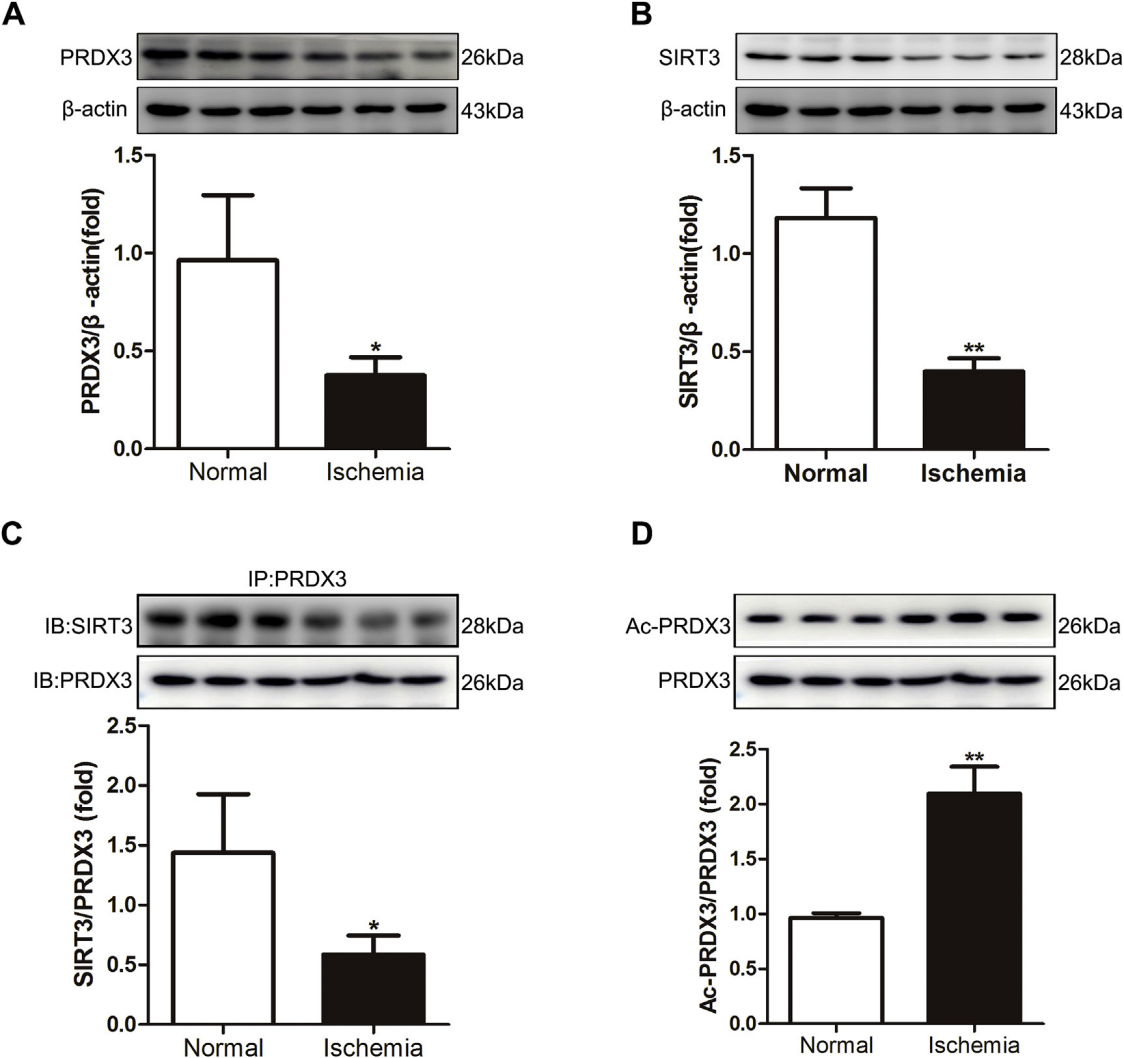
**Expression of SIRT3 and PRDX3 in the ischemic intestine of clinical patients**. (A) The expression of PRDX3 protein was normalized to β-actin expression in tissue lysates from the ischemic intestines of clinical patients, n = 3. (B) SIRT3 protein expression was normalized to β-actin expression in tissue lysates from the ischemic intestines of clinical patients, n = 3. (C) CoIP of PRDX3 with SIRT3. The input represents the total protein extract used in IP. IB, immunoblotting; IP, immunoprecipitation, n = 3. (D) PRDX3 acetylation, n = 3. *p < 0.05, **p < 0.01.

We then examined the expression of PRDX3 and SIRT3 in the ischemic intestine of clinical patients. The expression of PRDX3 and SIRT3 in ischemic intestinal tissue was lower than that in normal tissue (Fig. 8A-B). The binding of SIRT3 with PRDX3 was also decreased in the ischemic intestine (Fig. 8C), and correspondingly, the acetylation level of PRDX3 was increased (Fig. 8D). These results are in accordance with those measured in mice with intestinal I/R injury.

## 4 Discussion

Intestinal I/R is a critical illness associated with high morbidity and mortality. Mitochondrial oxidative stress and cell apoptosis are the key pathogenic events resulting from intestinal I/R injury [5,[50], [51], [52]]. In this study, we first reported the following observations: (1) PRDX3 protects against intestinal I/R-induced mitochondrial oxidative damage and apoptosis. (2) The acetylation of PRDX3 inhibits its antioxidative activity in intestinal I/R injury. (3) SIRT3 deacetylates PRDX3 and can therefore alleviate intestinal I/R-induced mitochondrial oxidative damage and apoptosis.

Accumulated ROS in mitochondria, which mainly consist of ·O_2_^−^ and H_2_O_2_, are the dominant source of intracellular oxidative stress. Most of the ·O_2_^−^ generated by the mitochondria is released into the mitochondrial matrix, where it impairs mitochondrial function. Although ·O_2_^−^ cannot cross the mitochondrial membrane because of its negative charge [53,54], it can be converted to H_2_O_2_ by MnSOD in the mitochondria. Mitochondrial H_2_O_2_ readily diffuses through membranes to enhance a different type of oxidative stress and damage cellular macromolecules, such as proteins, lipids, and nucleic acids, particularly after its conversion to ·OH [55]. We confirmed that increased mitochondrial H_2_O_2_ and its subsequent deleterious effects play an important role in intestinal I/R injury; thus, mitochondrial H_2_O_2_ scavenging might be an effective strategy for attenuating I/R-induced ROS accumulation and apoptosis.

Noxious H_2_O_2_ is mainly removed by catalase, glutathione peroxidase (GPx) and members of the peroxiredoxin (PRDX) family [56], and PRDX3, GPx1 and GPx4 are the main members of this family located in mitochondria [57]. However, lower relative abundances of GPx1 and GPx4 limit their ability to compete with PRDX3 [20], which makes PRDX3 the target for almost all mitochondrial H_2_O_2_. Previous studies have shown that PRDX3 overexpression scavenges excess H_2_O_2_ and protects cells from H_2_O_2_-induced apoptosis [58]. Here, we first found that PRDX3 was decreased after intestinal I/R injury and in Caco-2 cells after H/R injury. Furthermore, the overexpression of PRDX3 in Caco-2 cells decreased the levels of mitochondrial oxidative damage and apoptosis induced by H/R, as indicated by reduced mitochondrial H_2_O_2_ and superoxide levels, the rescue of mitochondrial dysfunction and morphology, decreased cleaved-caspase 3 activity and fewer TUNEL-positive cells. Therefore, PRDX3 might be a new therapeutic target for intestinal I/R injury.

Because PRDX3 plays a vital role as an antioxidant in intestinal I/R injury, we further explored the mechanism through which PRDX3 regulates this process. Multiple PRDX3 posttranslational modifications, including acetylation [29,36], ubiquitination [59,60], malonylation [61] and succinylation [62,63], have been reported. In this study, we focused on the effect of deacetylation on PRDX3 activation. Protein acetylation regulates several cellular functions, such as oxidative stress, energy production, autophagy and cell death [64]. Here, the acetylation of PRDX3 was significantly increased in both *in vitro* and *in vivo* intestinal I/R models. Importantly, the inhibition of SIRTs by NAM increased the acetylation of PRDX3 and impaired its capability to protect against mitochondrial oxidative and apoptosis. These results indicate that PRDX3 acetylation, which might be regulated by NAD^+^-dependent deacetylase, inhibits the activity of PRDX3.

SIRT3, SIRT4 and SIRT5 are the three members of the SIRT family located in mitochondria. Among these, SIRT3 is the major regulator of the mitochondrial acetylome and targets most mitochondrial proteins [29,65]. Many lines of evidence show the role of SIRT3 in mitochondrial homeostasis [66] and ROS management [67]. The protective effects of SIRT3 in mitochondria have been verified in some I/R models [[33], [34], [35]]. In this study, we first investigated the protective role of SIRT3 in intestinal I/R injury and the function of SIRT3-dependent deacetylation and activation of PRDX3. SIRT3 expression decreased in a time-dependent manner during intestinal I/R injury and in Caco-2 cells after H/R injury and was negatively correlated with PRDX3 acetylation. Moreover, SIRT3 directly binds and deacetylates PRDX3, as demonstrated through coIP and IP experiments. More importantly, NAM cannot increase PRDX3 acetylation in SIRT3-knockdown experiments. These results indicate that SIRT3 is the direct NAD^+^-dependent deacetylase that deacetylates and increases the activity of PRDX3; however, the detailed mechanism of this deacetylation needs to be further elucidated.

Previous high-throughput human proteomic assessments have shown that SIRT3 can deacetylate PRDX3 by targeting the lysine residues K83, K91, K253 or K196 on PRDX3 [29,36]. By screening these four potential acetylation sites in Caco-2 cells, we identified K253 as the site of PRDX3 acetylation. Functionally, PRDX3 deacetylation at K253 by SIRT3 increased the dimerization of PRDX3 in Caco-2 cells after subjected to 12 h hypoxia and followed 4 h reoxygenation, and this effect was accompanied by decreases in the levels of mitochondrial damage and apoptosis induced by H/R injury in Caco-2 cells. In contrast, the simulation of acetylation by PRDX3-K253Q decreased its dimerization and abolished the protective effect of PRDX3 overexpression in Caco-2 cell H/R injury. Thus, the K253 residue might be the dominant functional acetylation site on PRDX3 that is regulated by SIRT3 in Caco-2 cells.

In summary, our study provides the first confirmation of the important role of PRDX3 in intestinal I/R injury. Furthermore, the results show that the SIRT3-mediated deacetylation of PRDX3 on residue K253 increases the activity of PRDX3 in Caco-2 cells after H/R injury. These findings identify SIRT3/PRDX3 as a critical signaling pathway in intestinal I/R injury and provide a new therapeutic target for intestinal I/R injury.

## Declaration of competing interest

The authors have declared that no competing interest exists.

## Abbreviations

PRDX3: peroxiredoxins3
I/R: ischemia/reperfusion
SIRT3: sirtuin-3
MODS: multiple organ dysfunction syndrome
SIRS: systemic inflammatory response syndrome
H_2_O_2_: hydrogen peroxide
ROS: reactive oxygen species
·OH: hydroxyl radical
NAD+: nicotinamide adenine dinucleotide
CypD: cyclophilin D
MnSOD: manganese superoxide dismutase
IDH2: isocitrate dehydrogenase
H&E: hematoxylin and eosin
H/R: hypoxia/reoxygenation
IP: immunoprecipitation
coIP: coimmunoprecipitation
HDAC: histone deacetylase
GPx: glutathione peroxidase
NAM: nicotinamide
TSA: trichostatin A

## Footnotes

Appendix A Supplementary data to this article can be found online at https://doi.org/10.1016/j.redox.2019.101343.
