# Supplementary material for: SIRT3-mediated deacetylation of PRDX3 alleviates mitochondrial oxidative damage and apoptosis induced by intestinal ischemia/reperfusion injury
Source: Redox Biol. 2019 Oct 12;28:101343. doi: 10.1016/j.redox.2019.101343 (PMC6820261; doi:10.1016/j.redox.2019.101343)
Supplement: Multimedia component 1 [file mmc1.docx]

**Supplementary Material**

**Table 1 Potential acetylation sites of PRDX3 in humans and mice.**

| Lysine residue | Species | Amino acid sequence |
| --- | --- | --- |
| K83 | Homo sapiens | VVNGEFKDLSLDDF |
|  | Mus musculus | VVNGEFKELSLDDF |
| K91 | Homo sapiens | DDFKGKYLVLFFYP |
|  | Mus musculus | DDFKGKYLVLFFYP |
| K196 | Homo sapiens | PNGVIKHLSVNDLP |
|  | Mus musculus | PNGVVKHLSVNDLP |
| K253 | Homo sapiens | PSPAASKEYFQKVNQ |
|  | Mus musculus | PSPTASKEYFEKVHQ |

**Supplementary Figure 1 SIRT3 protects against intestinal I/R injury *in vitro*.**

(A) SIRT3 protein expression was normalized to β-actin expression in total cell lysates from Caco-2 cells, n=6. ***p*<0.01 compared with the Sham group. (B-H) Caco-2 cells were transfected with the SIRT3 expression plasmid or vector plasmid for 48 h and were then subjected to 12 h of hypoxia followed by 4 h of reoxygenation to achieve H/R. (B) SIRT3 protein expression was increased after transfection with the SIRT3 expression plasmid, n=3. (C) PRDX3 acetylation, n=3. (D) Mitochondrial H_2_O_2_ level, n=8. (E) MitoSOX Red and flow cytometry analysis of cells stained with MitoSOX dye. Scale bar=25 μm, n=6. (F) Caspase-3 activity in Caco-2 cells, n=8. (G) Cleaved caspase-3 protein expression was normalized to β-actin expression in total cell lysates from Caco-2 cells, n=3. (H) TUNEL and DAPI staining. The apoptotic index is represented as the ratio of TUNEL-positive cells to DAPI staining. Scale bar=50 μm, n=6. **p*<0.05, ***p*<0.01.

**Supplementary Figure 2 H_2_O_2_ causes dimerization of PRDX3**

(A) Caco-2 cells were divided into two groups, control group and H_2_O_2_ group. Caco-2 cells in H_2_O_2_ group were treated with 1mM H_2_O_2_ for 15 min. Representative western blot of PRDX3 monomer and dimer in Caco-2 cells, n=3. ***p*<0.01 compared with control group.
